# Work Practices and Health Problems of Spray Painters Exposed to Organic Solvents in Ile-Ife, Nigeria

**DOI:** 10.5696/2156-9614-10.28.201208

**Published:** 2020-12-02

**Authors:** Temitope Olumuyiwa Ojo, Adedeji Ayodeji Onayade, Olusegun Temitope Afolabi, Macellina Yinyinade Ijadunola, Oluwaseun Taiwo Esan, Patrick Ayodeji Akinyemi, Oluwaseun Olaniyi Awe

**Affiliations:** 1 Department of Community Health, Faculty of Clinical Sciences, Obafemi Awolowo University, Ile-Ife, Nigeria; 2 Department of Community Health, Obafemi Awolowo University Teaching Hospitals Complex, Ile-Ife, Nigeria; 3 Department of Ophthalmology, Faculty of Clinical Sciences, Obafemi Awolowo University, Ile-Ife, Nigeria

**Keywords:** spray painters, organic solvents, Nigeria

## Abstract

**Background.:**

Automobile spray painters in Nigeria are exposed to organic solvents due to the hazardous nature of their work. Inadequate use of personal protective equipment (PPE) may intensify exposure to high levels of chemical hazards with resultant health problems.

**Objectives.:**

The present study assessed PPE use and work practices and compared work-related health problems of spray painters and controls in Ile-Ife, Nigeria.

**Methods.:**

A cross-sectional study was conducted among 120 spray painters and 120 controls (electronic technicians). Data on socio-demographics, work practices, knowledge about organic solvent-related hazards and self-reported health symptoms were obtained using a semi-structured questionnaire. Clinical examinations were performed for all respondents and the composition of organic solvents in paints and paint products were derived from material safety data sheets.

**Results.:**

All respondents were male, and the mean age was 32.7±13.8 years for painters and 33.9±15.5 years for controls. Few (7.5%) painters perceived their use of PPE to be adequate. All spray painters worked in enclosed workshops and N-butyl acetate was the most commonly used organic solvent. Spray painters reported excessive tear production, recurrent cough, and short-term memory loss more frequently than controls (P<0.05). In addition, 89% of painters noticed paint-stained sputum immediately after spray painting. The prevalence ratio of respiratory symptoms was higher in spray painters than controls (prevalence ratio=21.0, CI=2.9–153.6). On clinical examination, more spray painters had corneal opacity and dry skin when compared with controls (P<0.05).

**Conclusions.:**

Spray painters in the study area worked amidst chemical hazards and had poor use of PPE. Exposure to organic solvents may be responsible for the higher prevalence of self-reported health problems among spray painters. Interventions to enforce the use of PPE and improve the knowledge of organic solvent-related hazards among spray painters are essential.

**Participant Consent.:**

Obtained

**Ethics Approval.:**

Ethical approval to conduct the study was obtained from the Health Research and Ethics Committee of the Institute of Public Health, Obafemi Awolowo University, Ile-Ife Nigeria (HREC No: IPHOAU/12/463).

**Competing Interests.:**

The authors declare no competing financial interests.

## Introduction

Spray painting is an occupation characterized by exposure to volatile organic compounds (VOCs) during mixing, painting, drying and cleaning of paints.^1^ This exposure may be intensified when painting and allied activities are performed without the use of personal protective equipment (PPE) such as gloves, aprons or respirator masks.^2^ In Nigeria, spray painters may have high VOC exposures because most auto spray painting in Nigeria is done in workshops and sheds that do not have specialized spraying booths. A typical spraying workshop is a basic wooden structure with three walls and an opening in front, and a store at the rear. Tarpaulin or nylon material is commonly used as retractable covering for workshop openings before and during the painting process, a situation that reduces dilution ventilation and increases workers' exposure to VOCs. This open spraying method also exposes the immediate environment around the workplaces to an extremely high level of chemical hazards.^3^

Exposure to VOCs may have deleterious health effects. Volatile organic compounds are potent irritants to the eyes as well as the mucous membranes result in excessive tearing and red eyes. Allergenic components of paints may also result in red itchy eyes among some workers.^4^ Dermatological effects of exposure to VOCs may include dermatitis—both irritant and contact, as well as excessively dry skin. In addition, VOCs may affect the liver, manifesting as hepatitis and raised liver enzymes.^5^ Other effects may include headaches, anosmia, paresthesia, impaired memory and reaction time and changes in sense of balance.^6^ Chronic exposure leads to damage to the central nervous system and kidneys, with increased risk of cancers. Reproductive and developmental effects in exposed pregnant women include spontaneous abortion, skeletal abnormalities and delivery of low birth weight babies.^7,8^ Decreased lung function parameters and high prevalence of asthma symptoms have also been documented in previous studies.^9,10^ Most studies assessing the health effects of exposure to VOCs are primarily from developed countries with strict workplace safety standards and enforcement. As a result, the effects of VOC exposure in developing countries remain understudied.

A major occupational health challenge in developing countries is to ensure that workers in the informal sector have access to basic occupational health services and health promotion efforts. This is essential to enable workers to adopt safe work practices against a backdrop of low working capital. The situation in Nigeria is similar with regards to artisans, particularly spray painters. Nigerian spray painters operate in the small and medium enterprise (SME) sector of the economy, and SMEs are a major contributor to the Nigerian economy.^11,12^ However, there is limited policy intervention to ensure that artisans in this sector operate in safe work settings. Moreover, Nigeria has a weak and poorly organized health surveillance system for early recognition and prompt management of occupational diseases among artisans who work in SMEs.^13^ Hence, the enforcement of safety regulations, inspection of work premises and the promotion of appropriate use of PPE among artisans in SMEs are inadequate.

**Abbreviations**
*FEV1*: Forced expiratory volume in the first second
*FVC*: Forced vital capacity
*PPE*: Personal protective equipment
*SME*: Small and medium enterprises
*VOCs*: Volatile organic compounds
*WHO*: World Health Organization

Furthermore, the prevalence of adverse health effects due to use of organic solvent use among automobile spray painters in Nigeria is poorly documented. The few existing studies among spray painters have focused on lung function, biochemical profile or a questionnaire-based assessment of knowledge about work hazards in isolation.^14,15^ Thus, the present study assessed spray painters' knowledge of hazards associated with organic solvent use, availability and use of PPE and self-reported health problems. The findings of this study will guide the formation of policy needed to enforce workplace hazard monitoring and control. The study's findings may aid appropriate interventions necessary to improve the knowledge of these artisans about the hazards of their occupation and promote healthy work practices.

## Methods

This survey was carried out in Ile-Ife, a semi-urban town in southwest Nigeria, whose inhabitants are mostly civil servants, farmers, traders, and artisans.

### Study design/population

The present study used a cross-sectional analytic design. The study group was comprised of 120 spray painters (60 master painters and 60 apprentice painters) selected from a sampling frame of 96 master spray painters and 76 eligible apprentices via the simple random sampling technique. For the controls, 120 subjects (60 master electronic technicians and apprentices each) were selected by matching the characteristics of the selected spray painters from a sampling frame of 158 master electronic technicians and 92 eligible apprentices. The study participants in both study and control groups were matched for sex, age, weight and height. All participants were male as the two vocations are male dominated.

### Data collection

Semi-structured interviewer-administered questionnaires were used for data collection, and some questions which included open and closed questions were adapted from the American Thoracic Society's recommended respiratory disease questionnaire for use in epidemiological research.^16^ A clinical examination proforma was used to obtain respondent biomedical data such as weight, height and blood pressure. Information was obtained on socio-demographics, knowledge about health effects of organic solvents in spray paints, general adverse health effects due to the use of spray paints, as well as the availability and use of PPE.

Spray painters were also asked to name the chemical products (paints, thinners and lacquer) that they commonly used. A list of common organic solvents was derived from the material safety data sheet of these products. Questions from the short form of the Mini-Mental State Examination scale were used to assess short term memory.^17^ The interviewer named three non-related objects for one second each. Respondents were then asked to name the objects. One mark was awarded for each correct answer (maximum of 3 marks). The names of the objects were repeated until the respondents learned all three. After about 5 minutes, respondents were then asked to recall the names of the objects. To derive scores for short term memory, one mark was awarded for each correct answer to the questions on memory. In total, a maximum score of 6 was obtainable and individuals with a score of less than 4 were considered to have recall deficit as specified by the short form of the Mini-Mental State Examination scale. The questionnaire was translated into Yoruba, which is the local language in the study area.

A walk through inspection of the workplaces of all spray painters in this study was also conducted and a checklist was completed to document the type of solvent control measures in use and the nature of available PPE. For this study, ambient concentration of organic solvents was assessed in all artisan workshops using the E8500 plus (E instruments, Philadelphia, USA). The details of air sampling were reported elsewhere.^18^ Data were collected between October to December 2016.

### Clinical examinations

Physical examination of study participants was conducted privately in their workshops. All clinical examinations were conducted by trained physicians. Measurement of standing height was done with the aid of a stadiometer which comprised a horizontal platform, vertical perpendicular rule and a sliding head-board. Heights were taken in a standardized approach with participants' shoes removed and heels together and toes pointed slightly out, standing chest out and erect with their backs towards the vertical rule and their heels, buttocks and occiput touching the rule simultaneously with participants looking horizontally straight ahead. The investigator's eyes were placed level with the headboard to avoid parallax error. Measurements were recorded to the nearest centimeter after sliding down the headboard. All study participants were weighed wearing light clothing and barefooted. Weights were measured using a Camry® weighing scale (model BR9012, Zhongshan Camry Electronic Co. Ltd, Guangdong, China). The scale was standardized daily before use and measurements were considered to the nearest decimal point of 0.5 kg.

Blood pressure was measured using an Accuson's mercury sphygmomanometer (Kris-alloy, England). Blood pressure was measured in the sitting position with an appropriate cuff size on the left arm. Two consecutive measurements were obtained 5 minutes apart for each participant and the average blood pressure was recorded. Systolic blood pressure was recorded at phase 1 of Korotkoff sounds while diastolic blood pressure was recorded at phase V Korotkoff sounds. A blood pressure greater than 140/90 mm mercury after two separate readings was regarded as elevated.

Visual acuity of each eye was assesed in succession (right eye first) by trained research assistants with a 3-meter Snellen acuity chart or Tumbling ‘E' chart (for non-literate participants). Visual acuity was done in an environment with natural illumination that was free from sun glare. Artisans were instructed to wash their hands and carefully occlude the non-tested eye with the ipsilateral palm while avoiding pressure on the eyeball during visual acuity testing. Participants stood at a distance of 3 m (10 feet) and were told to start reading from the top of the chart. Visual acuity was expressed as the notation of the smallest line of optoypes read as indicated on the chart. When an artisan was unable to identify the largest optotype on the chart, the visual acuity was determined by the ability to count fingers at 3 m, hand motion, perception of a point light source or no light perception. A visual acuity of worse than 6/18 was regarded as visual impairment based on the World Health Organization (WHO) International Classification of Diseases.^19^ All participants had their eyes examined with the aid of a pen torch and handheld magnifying lens to identify abnormalities of the lid, conjunctiva and cornea, such as conjunctiva hyperemia and corneal opacity. Participants were reported to have corneal opacity if there was loss of transparency of any part of the cornea causing partial or total obscuration of the details of the underlying iris and/or pupil.

Spirometry was conducted using the Spirolab III® (MIR, Italy) device. Participants were made to stand relaxed in front of the apparatus without using a nose clip and with no tight clothing. The procedure was explained to participants. Subsequently, measurements of forced vital capacity (FVC) and forced expiratory volume in the first second (FEV_1_) were obtained. The best readings of FVC and FEV_1_ were taken from three technically satisfactory forced expiratory maneuvers. Corrections for body temperature and pressure saturated with water vapor were automatically made for all measurements by the device software.

### Data analysis

Data were analyzed using the Statistical Package for Social Sciences (SPSS) version 20.0. Continuous variables such as age, height and income were summarized using mean and SD, while categorical variables were summarized using frequencies and proportions. Student's t-test was used to assess difference in means across the study and control groups. Chi-square test was used to assess the relationship between categorical variables e.g. smoking status and abnormal spirometry results.

Participants' knowledge about organic solvent-related health problems and PPE that should be used during painting was assessed and graded. Each correct answer had a score of one while wrong answers were scored zero. There were 8 questions about organic solvent-related health problems and an individual with a cumulative score of at least 4 (50%) was categorized as having good knowledge, while individuals who scored less than 4 were classified as having poor knowledge. There were 9 questions about PPE that should be used during painting. An individual with a cumulative score of at least 5 was categorized as having good knowledge, while individuals who scored less than 5 were classified as having poor knowledge.

Likelihood ratio was used to assess the relationship between categorical variables when more than 25% of the cells had an expected count less than 5. Prevalence ratio (alongside 95% CI) was used as a measure of effect among the study groups. A *P*-value of < 0.05 was taken as statistically significant.

### Ethics

Ethical approval to conduct the study was obtained from the Health Research and Ethics Committee of the Institute of Public Health, Obafemi Awolowo University, Ile-Ife Nigeria (HREC No: IPHOAU/12/463). Written consent was obtained from all adult participants. Assent was obtained from apprentices less than 18 years and permission was obtained from their parents or guardians.

## Results

One hundred and twenty spray painters and one hundred and twenty electronic technicians (controls) were recruited into the study. There was no significant difference in the age, height and weight of spray painters and controls. The organic solvents most commonly used by spray painters are shown in Table 1. N-butyl acetate was the solvent with the highest percentage by weight (25–50%) among the paints commonly used by spray painters in Ile-Ife. Ethanol had the highest percentage by weight (50–100%) among the major components of thinners used by spray painters. N-butyl acetate also comprised 20–35% by weight of the most common lacquers used by spray painters in Ile-Ife.

**Table 1:** Composition of Organic Solvents in Paints, Thinners and Lacquers Commonly used by Spray Painters in Ile-Ife

Commonly used organic solvents*	**Percentage by weight**
Major components - paints	
N-butyl acetate	25–50
Xylene	10–25
Ethyl benzene	2.5–10
Butanol	2.5–10
Acetone alcohol	1–2.5
Major components - thinners	
Ethanol	50–100
N-butyl acetate	10–25
Isobutyl acetate	10–25
Methanol	0–2.5
Major components - lacquers	
N-butyl acetate	20–35
Xylene	10–25
Toluene	10–15
Acetone	10–15
Ethyl benzene	5–10

^*^ Derived from appropriate Safety Data Sheets of the products

Knowledge of health hazards due to organic solvents was generally low among spray painters. Only 30% of spray painters knew that exposure to organic solvents may be associated with headaches, while 38 (31.7%) painters indicated that organic solvent exposure may be associated with breathing problems. Fifty-nine (49.2%) spray painters indicated that organic solvent exposure may be associated with eye damage, while only 29 (24.2%) correctly indicated that exposure to organic solvents may be associated with damage to internal organs. Only seven (5.8%) spray painters correctly identified that exposure to organic solvents may be associated with cancers *(Table 2).*

**Table 2:** Knowledge of Health Hazards Due to Solvent Exposure Among Spray Painters

**Knowledge of health hazards**	**Frequency (n=120)**	**Percentage**
Eye damage	59	49.2
Recurrent cough	56	46.7
Recurrent catarrh	51	42.5
Breathing problems	38	31.7
Recurrent headaches	36	30.0
Damage to internal organs	29	24.2
Skin rash	23	19.2
May be associated with cancer	7	5.8
Overall knowledge about organic solvent-related health problems		
Good	41	34.2
Poor	79	65.8

Most (92.5%) spray painters indicated that a nose (dust) mask should be used as PPE during spray painting. Seventy-nine (65.8%) spray painters correctly indicated that safety boots should be used as PPE, while 74.2% reported that waterproof overalls should be used as PPE for painters. In addition, 71 (59.2%) spray painters stated that waterproof hand gloves are appropriate PPE. Only 34 (28.3%) spray painters identified safety goggles as appropriate PPE for painting, while only 18 (15%) stated that respirators should be used as PPE for spray painters *(Table 3).*

**Table 3:** Knowledge of Personal Protective Equipment Recommended During Spray Painting

**Characteristics**	**Frequency (n=120)**	**Percentage**
Nose mask (dust)	111	92.5
Waterproof overalls	89	74.2
Head cover	81	67.5
Safety boots	79	65.8
Waterproof hand gloves	71	59.2
Face shield	52	43.3
Safety goggles	34	28.3
Respirators	18	15.0
Hearing protector	0	0
Overall knowledge about PPE		
Good	83	69.2
Poor	37	30.8

The use of PPE among spray painters is summarized in Table 4. None of the spray painters used safety goggles, respirator with an airline, face shield or hearing protector while painting. Twenty-eight (23.3%) painters used nose masks and only five (4.2%) used waterproof overalls while painting. Almost all (92.5%) spray painters perceived their self-use of PPE as inadequate. The most common reason (33.3%) for non-use of PPE by individual spray painters was that they were not readily available.

**Table 4:** Use of Personal Protective Equipment by Spray Painters

**Characteristics**	**Frequency (N=120)**	**Percentage**
Use nose (dust) mask	28	23.3
Use safety boots	21	17.5
Use head cover	15	12.5
Use hand gloves	8	6.7
Use waterproof overalls	5	4.2
Use respirator with filter	3	2.5
Perceived own PPE use as adequate	9	7.5
Reason for non-use of PPE*		
Not readily available	40	33.3
Too costly	37	30.8
Not comfortable to use	25	20.8
Ignorance	22	19.8
Negligence	10	9.0
Not necessary	9	8.1
†Not applicable	9	
Perceived PPE use by other spray painters as adequate	0	0.0
Perceived reason for non-use of PPE by other spray painters*		
Not readily available	46	38.3
Too costly	34	28.3
Ignorance	34	28.3
Not comfortable to use	30	25.0
Negligence	30	25.0
Not necessary	15	12.5
Inappropriate respiratory protective equipment used by spray painters		
Surgical mask	23	19.2
Cloth shield over the nose	12	10.0
Cotton wool nose plug	10	8.3
Foam nose plug	9	7.5

*Multiple responses allowed

†Not included in the analysis

Thirty-seven (30.8%) spray painters indicated that high procurement cost was a reason for non-use of PPE. However, nine (8.1%) painters attributed their non-use of PPE to the fact that it was not necessary. All spray painters perceived that their fellow painters do not use PPE as adequately as they should. The most common reason for this was that PPE were not readily available (38.3%). Only 15 (12.5%) spray painters believed that the reason for non-use of PPE by fellow painters was because it was not necessary. Surgical face masks, which are inappropriate respiratory protective equipment, were reported to be used by 23 (19.2%) spray painters while painting. Other inappropriate items used as respiratory PPE included cotton wool and foam nose plugs as well as tying cloth over the nose.

The distribution of self-reported health problems by study participants is presented in Table 5. Excessive eye tear production (irritant-induced lacrimation), recurrent cough, short term memory loss and chest pain occurred more among spray painters than in electronic technicians (*P* < 0.05). However, anxiety was more common among electronic technicians than spray painters and this was statistically significant (*P*=0.028). All other symptoms such as reduced vision, eye itching, skin itch, skin irritation, recurrent headaches and insomnia were not significantly different among spray painters and electronic technicians. The prevalence of respiratory symptoms was 21 times higher in spray painter than electronic technicians (CI=2.9–153.6).

**Table 5:** Prevalence Ratios of Self-Reported Health Symptoms (N=120 in Each Artisan Group)

**Health problems**	**Painters N (%)**	**Electronic technicians N (%)**	**Statistical indices**
**Eye symptoms**			
Reduced vision	21 (17.5)	18 (15.0)	χ^2^=0.3, *P*=0.600
Eye itching	16 (13.3)	14 (11.7)	χ^2^=0.2, *P*=0.696
Excessive tear production	13 (10.8)	4 (3.3)	χ^2^=5.1, *P***=0.024**
Red eyes	7 (5.8)	2 (1.7)	LR=3.1, *P*=0.081
Eye pain	5 (95.8)	2 (1.7)	LR=1.4, *P*=0.242
**Respiratory symptoms**			
Recurrent cough	11 (5.8)	0 (0.0)	χ^2^=11.5, *P***=0.001**
Chest wheeze	9 (7.5)	1 (0.8)	χ^2^=6.7, *P***=0.010**
Wheezing with breathlessness	7 (5.8)	0 (0.0)	LR=9.9, *P***=0.002**
Chest pain	9 (7.5)	0 (0.0)	LR=12.8, *P***<0.001**
**Dermatologic symptoms**			
Skin rash	5 (4.2)	4 (3.3)	LR=0.1, *P*=0.734
Skin itch	10 (8.3)	6 (5.0)	χ^2^=1.1, *P*=0.301
**Neurologic symptoms**			
Recurrent headaches	3 (2.5)	8 (6.7)	χ^2^=2.4, *P*=0.123
Short term memory loss	19 (15.8)	8 (6.7)	χ^2^=5.1, *P***=0.025**
Dizziness	4 (3.3)	1 (0.8)	*LR=1.8, *P*=0.161
Anxiety	3 (2.5)	11 (9.2)	χ^2^=4.9, *P***=0.028**
Insomnia	9 (7.5)	4 (3.3)	χ^2^=2.0, *P*=0.154
At least one eye symptom	43 (35.8)	29 (24.2)	PR=1.483, *CI*=1.0–2.2
At least one respiratory symptom	21 (17.5)	1 (0.8)	*PR=21.0*, **CI=2.9–153.6**
At least one dermatological symptom	11 (9.2)	6 (5.0)	PR=1.8, CI=0.7–4.8
At least one neurological symptom	28 (23.3)	21 (17.5)	PR=1.3, CI=0.8–2.2

Abbreviations: LR, likelihood ratio; PR, prevalence ratio.

Corneal opacity, immediate recall deficit and dry skin were more common among spray painters than controls and this was statistically significant (*P* < 0.05). Other clinical findings were not significantly different among spray painters and electronic technicians (*P* > 0.05). Only one master spray painter had mixed (both obstructive and restrictive) ventilatory impairment. The prevalence of respiratory impairment was 14 times higher in spray painters than in electronic technicians (CI=1.9–104.8) *(Table 6).*

**Table 6:** Health Problems on Physical Examination by Study Participants

**Health problems**	**Painters N (%)**	**Electronic technicians N (%)**	**Statistical indices**
Red eyes	5 (4.2)	8 (6.7)	χ^2^=0.7, *P*=0.392
Visual impairment	9 (7.5)	5 (4.2)	χ^2^=1.2, *P*=0.271
Corneal opacity	3 (2.5)	0 (0)	LR=4.2, *P***=0.040**
Dry skin	7 (5.8)	0 (0)	LR=9.9, *P***=0.002**
Dermatitis	3 (2.5)	3 (2.5)	LR=0.0, *P*=1.000
Obstructive impairment on spirometry	10 (8.3)	1 (0.8)	χ^2^=7.7, *P***=0.005**
Restrictive impairment on spirometry	6 (5)	0 (0)	LR=8.5, *P***=0.004**
Elevated blood pressure	12 (10.0)	5 (4.2)	χ^2^=3.1, p=0.078
Immediate recall deficit	10 (8.3)	0 (0)	χ^2^=10.4, *P***=0.001**
At least one ocular sign is present	14 (11.7)	13 (10.8)	PR=1.1, CI=0.5–2.2
At least one dermatological sigh is present	10 (8.3)	3 (2.5)	PR=3.3, CI=0.9–11.8
At least one type of pulmonary function impairment is present	14 (11.7)	1 (0.8)	PR=14.0, **CI=1.9–104.8**

Abbreviations: LR, likelihood ratio; PR, prevalence ratio. N=120 in each artisan group.

## Discussion

Spray painters work under sub-optimal conditions because of poor capital outlay to establish standard spraying booths. This exposes them to high levels of chemical hazards. The present study assessed PPE use, knowledge of health hazards and occurrence of health-related symptoms and signs among spray painters exposed to organic solvents in Ile-Ife, Nigeria. Our findings support the hypothesis that the health status of painters exposed to organic solvents differs significantly from that of non-exposed electronic technicians. As published elsewhere, the exposure of spray painters in this study to organic solvents during spray painting (expressed as an 8-hour weighted average of total exposure to VOCs) was 13.4 ppm, which was substantially above the national permissible exposure limit of 1.9 ppm.^18,20^

In the present study, N-butyl acetate was the organic solvent most commonly used, according to average percentage by weight as identified in the product material safety data sheets. N-butyl acetate*,* a carboxylic acid ester that is a colorless solvent with a fruity odor, has been documented to irritate the eyes, skin, nose, respiratory tract and central nervous system in humans.^21^ Ethanol and xylene were the second and third most common organic solvents used by spray painters in Ile-Ife. Ethanol and xylene have been implicated in causing central nervous system depression with manifestations such as dizziness, headache, nausea and vomiting.^22^ Our findings differ from the results of a nationwide workplace survey in Japan, where toluene (an aromatic hydrocarbon) was the most common solvent and N-butyl acetate was the fourth most common.^23^ Similarly, a survey of an industrial complex in South Korea found toluene to be the common organic solvent in use.^24^ The Korean study, however, assessed use of common organic solvents in a diverse work setting that included auto spray painters, furniture makers, and auto technicians, among others. Toluene has health effects resembling those of N-butyl acetate but is generally more toxic.

Toluene-based paints are often preferred because they have better aesthetics, produce a shinier finish, and evaporate faster, with a better drying effect. Besides the generally greater expense of toluene-based paint products, toluene may not be commonly used by spray painters in this study because, owing to recent concerns about safety and toxicity, regulatory agencies have mandated manufacturers to reduce concentrations of aromatic hydrocarbons in paints.^25^ In an industrial district in Beijing, China, 1-,2,4- trimethyl, benzene (124TMbenzene) was the organic solvent most commonly used in auto spraying workshops over a 5-year period.^26^ In Ile-Ife, 124TMbenzene was not identified as a solvent in use because it was not a component listed in any of the material safety data sheets on the paints, lacquers and thinners commonly used by spray painters evaluated in this study. Most paint manufacturers are now embracing other alternatives to benzene compounds and are therefore constrained to using derivatives of benzene in lower quantities.^25^

In the present study, painters generally had little knowledge of health hazards associated with organic solvent exposure during spray painting. About half of the spray painters knew that organic solvents in paints can damage the eye. This lack of knowledge has serious health implications, as ignorance about the harmful effects of organic solvents may predispose painters to mishandle paints and paint products. Especially worrisome is the fact that only about one-third of painters could correctly identify solvent exposure as a possible cause of recurrent headaches, breathing problems and damage to internal organs. A factor that may help explain the lack of knowledge about solvent-related health hazards among spray painters is the low level of formal education. About half of the spray painters had attended only primary school.

None of the spray painters stated that hearing protection should be worn during painting, an indication that the spray painters were unfamiliar with hearing protectors and their effectiveness as PPE. Most painters identified dust masks as necessary PPE during spray painting, but only a few (15%) indicated that respirators are essential PPE during painting. Thus, spray painters were insufficiently aware of the PPE that is needed during spray painting, as dust masks cannot filter out vaporized organic solvents and so offer no protection against solvent inhalation. It has been documented that the respirator is the most effective PPE for reducing inhalational solvent exposure among spray painters.^27^

Similarly, only one in four painters indicated that safety goggles should be worn during painting, and only two out of five reported that face shields should be worn. The PPE that were mentioned most frequently by the spray painters were the ones that could prevent dermal exposures; these included safety boots, head coverings water-proof overalls and gloves. The findings suggest that efforts to improve spray painters' knowledge of effective PPE should focus on respirators, safety goggles, hearing protectors and face shields.

Most painters perceived their self-use of PPE as inadequate, and all perceived their colleagues' use of PPE as inadequate. None reported using safety goggles, face shields or hearing protectors during painting. Very few used respirators with filters or wore gloves, and only one in five painters wore dust masks. These findings are similar to those of a study in Ghana where the majority of spray painters did not use PPE.^28^ The major reasons mentioned by painters for non-use of PPE in the present study were that PPE were not readily available and were costly, and that workers were not well informed about them and found them uncomfortable. These issues have been documented in previous studies.^28–30^

Some spray painters in the present study used other items in place of standard PPE. Notable among these makeshift items was the surgical face mask. Two in five painters who improvised wore surgical face masks, which cannot filter the organic solvents in the air. Most painters used the same surgical mask repeatedly, and many workshops were replete with dirty face masks, which is evidence of repeated use. Other items used in place of standard PPE were foam nose plugs, cotton wool nose plugs and cloth tied over the nose. All these non-standard items offer inadequate protection.^27^

Among symptoms associated with work exposure, eye symptoms were most commonly reported by respondents. Similarly, in a South African study, eye problems were the most common self-reported symptoms of painters.^31^ Reduced vision was reported by 17.5% of painters. In contrast, a study of paint factory workers in Lagos, Nigeria, with presumed organic solvent exposure found that 1.25% of workers had poor vision.^29^ This difference may reflect the fact that the paint factory workers were younger, with most having less than 8 years of work experience. Itching of the eyes was reported by 13.3% of painters; this percentage is much lower than the 55% reported in a study of spray painters in South Africa.^31^ This difference may be explained by the fact that most spray painters in Ile-Ife spend less than 20% of their work time on spray painting. However, the prevalence of eye itching in our study was much higher than the 6.25% reported among paint manufacturing factory workers in Lagos.^29^ The possibility that a paint manufacturing factory is a more formal workplace, with probable lower solvent exposure and better environmental controls and enforcement of the use of PPE among workers, may explain this difference.

Neurological symptoms were also common among the painters in the present study. Short term memory loss was the most common symptom, with 15.8% of respondents affected. In contrast, headache was the most common neurological symptom (reported by 68%) in a group of Palestinian spray painters.^32^ The prevalence of short term memory loss in the present study (15.8%) was much higher than the 0.75% reported among paint factory workers in Lagos.^29^ The younger age of the factory workers and fewer years of experience on the job may explain this difference. However, the prevalence in this study was lower than the 38% reported among spray painters in Palestine.^32^

In the present study, recurrent cough was reported by 5.8% of painters. This percentage is much lower than the 50% reported among painters in the study in Palestine, but most of the painters in the Palestinian study were cigarette smokers.^32^ The prevalence of cough in our study also was lower than the 28% reported in a study of 70 spray painters in India.^33^ The fact that most spray painters in the present study spent less than 20% of their time at work on spray painting may account for this marked difference; also, the study in India was conducted in a single workshop. In the present study, one in three spray painters reported experiencing a chemical taste in their mouths when painting, and 89% noticed paint in their sputum immediately after painting. This finding is consistent with the fact that most spray painters in this study did not use PPE at all or that the PPE being used were ineffective in limiting inhalation of paint and organic solvent fumes.

The presence of paint in sputum immediately after painting further confirms that spray painters have unmitigated inhalational exposure to organic solvents in paints and paint products. Overall, spray painters had more respiratory symptoms which may be due to occupational exposure, similar to findings from a past study where spray painters had higher prevalence ratios of respiratory symptoms.^34^ Similarly, more spray painters had ventilatory impairment on spirometry which may further suggest that occupational exposure of spray painters may have more marked effects on the respiratory system.

Dry skin was more common among painters than among controls (*P*=0.002). This finding was similar to those from a study in Denmark where painters were significantly more affected by dry skin and eczema than controls.^35^ The increased prevalence of dry skin among spray painters may occur because some spray painters use organic solvent-based liquids such as thinners to wash their hands and feet after spray painting. Direct contact with organic solvents can dissolve the skin's protective barrier of oils, causing drying and chapping of the skin.^36^ Elevated blood pressure occurred more frequently among spray painters than controls, but this difference was not statistically significant (*P*=0.078). The modest sample size may explain why no significant difference was found, even though a marked variation existed among painters and controls. Chest pain was significantly more common among spray painters than electronic technicians (*P* < 0.001). A possible explanation is that the spray painters expend time and energy sanding the surfaces of vehicles before spraying, thus exerting some strain on their pectoral and upper limb muscles.

Immediate recall deficit was more prevalent among spray painters than controls (*P*=0.001). Similarly, previous studies have reported increased rates of impaired memory among spray painters.^37,38^ However, electronic technicians (controls) reported anxiety as a work-related symptom more frequently than spray painters (*P*=0.028). Several factors may explain this, such as the fact that the supply of electricity required by electronic technicians for repairs is erratic in the study location and this may cause anxiety. In addition, electronic technicians may worry about how to repair electronic devices that are new and sometimes complex and with which they are often not very conversant.

### Limitations

The present study has a few limitations. These include possible recall bias regarding symptoms; however, general physical examinations and clinical tests were conducted to minimize this bias. Comparisons with results of other studies conducted among spray painters was difficult since PPE and types of spray material used may vary. One shortcoming of studies assessing the effects of job exposures on health is the “healthy worker effect”, where those who have experienced severe ill-effects of solvent exposure may have left the vocation while healthier workers remain on the job. However, given the harsh economic conditions in the country and the difficulty of gaining other employment, this bias is thought to have been low. All respondents were males since the two study groups are male dominated, hence study findings cannot be generalized to females.

## Conclusions

Spray painters in the present study worked amidst chemical hazards and made poor use of PPE. Organic solvent exposure may be associated with adverse health outcomes since symptoms were significantly more common in spray painters with higher solvent exposures than controls. These symptoms included excessive tear production, cough, chest pain and short-term memory loss. Clinical examinations also showed that corneal opacity, dry skin and immediate recall deficits occurred more frequently among spray painters than controls. Spray painters sometimes used inappropriate items such as surgical face masks, cotton wool and foam nose plugs and cloths tied over the nose as a form of PPE. Future studies using qualitative methods are needed to better understand the factors influencing the use of PPE among spray painters.

Emphasis should be placed on ensuring that use of PPE is fully enforced in spray painting workshops.

Provision of standard PPE (through government-assisted supply schemes) to minimize organic solvent exposure among spray painters is an appropriate intervention to safeguard the health of this group of artisans. Spray painters should be continually educated about the hazards of organic solvents in paints they work with, and periodic medical examinations (at least yearly) are recommended to detect work-related health conditions in spray painters.
